# A standardised framework for classifying estimates of reproductive isolation

**DOI:** 10.1093/evlett/qrag023

**Published:** 2026-06-26

**Authors:** Jonah M Walker, Christopher R Cooney, Jonna Kulmuni, Konrad Lohse, Joana I Meier, Richard M Merrill, Elizabeth Scordato, Carole M Smadja, Sonal Singhal, Roger K Butlin, Sean Stankowski

**Affiliations:** Department of Zoology, University of Cambridge, Cambridge, United Kingdom; Tree of Life Programme, Wellcome Sanger Institute, Hinxton, Cambridgeshire, United Kingdom; Ecology and Evolutionary Biology, School of Bioscience, University of Sheffield, Sheffield, United Kingdom; Institute for Biodiversity and Ecosystem Dynamics, University of Amsterdam, Amsterdam, Netherlands; Organismal and Evolutionary Biology, University of Helsinki, Helsinki, Finland; Institute of Ecology and Evolution, University of Edinburgh, Ashworth Laboratories, Edinburgh, United Kingdom; Department of Zoology, University of Cambridge, Cambridge, United Kingdom; Tree of Life Programme, Wellcome Sanger Institute, Hinxton, Cambridgeshire, United Kingdom; Division of Evolutionary Biology, LMU Munich, Planegg-Martinsried, Germany; Department of Biological Sciences, California State Polytechnic University, PomonaUnited States; Institut des Sciences de l’Evolution de Montpellier (ISEM), Univ Montpellier, CNRS, IRD, Montpellier, France; Department of Biology, California State University—Dominguez Hills, Carson, United States; Ecology and Evolutionary Biology, School of Bioscience, University of Sheffield, Sheffield, United Kingdom; Department of Marine Sciences, University of Gothenburg, Tjärnö Marine Laboratory, Strömstad, Sweden; Department of Genetics, Evolution and Environment, University College London, LondonUnited Kingdom

**Keywords:** bio-ontology, reproductive barriers, speciation

## Abstract

Understanding reproductive isolation (RI) between lineages is a central goal of speciation research. While the strength of RI has been estimated across a broad range of taxa, synthesizing these data remains challenging, partially because we lack a common language for classifying and reporting RI estimates. Here, we present the *Reproductive Isolation Ontology* (RIO), a structured framework designed to standardize the classification of RI estimates across most sexually reproducing organisms. The RIO comprises 3 interrelated domains, each organized into a nested hierarchy of classification terms. We demonstrate the utility of this approach and call for researchers to adopt and expand the RIO to facilitate the integration of speciation research.

## Why we need a standard language for reproductive isolation

When two lineages meet—whether naturally or via human manipulation—their futures will be shaped by reproductive barriers between them. These barriers come in a diversity of forms, such as differences in habitat preference and reduced fitness of hybrids, all of which serve to reduce the production of fertile hybrid offspring between lineages. The total of these barriers comprises reproductive isolation (RI) (Sobel & Chen, 2014). As the strength of RI between lineages increases, gene flow is expected to decrease (Westram et al., 2022), thus further promoting lineage divergence. Because RI reflects both the extent of divergence between lineages and the likelihood of further divergence, quantifying RI has become central to studying speciation (Dobzhansky, 1937a; Sobel & Chen, 2014).

Most studies have explored the role of RI in speciation by estimating the strength of RI between pairs of closely related lineages. The resulting estimates are accompanied by metadata on the lineages (e.g., degree of ecological or genetic divergence) and a classification of the specific RI components measured (e.g., habitat preference, hybrid sterility). Comparative work has synthesized estimates of RI from these studies, attempting to uncover more general patterns about the tempo and mode of RI evolution and its role in species diversification. For example, studies have shown that intrinsic postzygotic barriers can be particularly effective in limiting gene flow early in speciation (Coughlan & Matute, 2020), that ecological divergence predicts the extent of RI (Funk et al., 2006), and that the rate of RI evolution does not predict macroevolutionary speciation rate variation in fruit flies or birds (Rabosky & Matute, 2013).

Comparative studies are still few in number and limited in scope, partially because comparative datasets on RI are scarce. Thus, it is unclear whether these findings will hold across more diverse sets of taxa, and many further questions about the role of RI in speciation remain unanswered. Filling these gaps requires datasets that compile standardized estimates of RI across large and diverse sets of taxa, but empirical estimates of RI are collected using multiple methods and approaches, at different levels of resolution, with terminology that varies across study systems (Stankowski et al., 2024). This endeavor is further complicated because most RI data have been published as standalone case studies focusing on a small number of lineages. Synthesizing across this vast and heterogeneous body of work requires (1) a standardized approach to *quantifying* the strength of RI components and (2) a standardized language for *classifying* these RI components to ensure the intended barriers are compared. A common language for both RI strength and classification will allow us to make comparisons across different study designs and taxonomic groups to better understand how RI evolves. Our field already has methods that allow RI strength to be quantified from empirical data in a way that is comparable across diverse taxa (e.g., the RI_4_ index; Sobel & Chen, 2014). However, while various authors have developed their own schema to classify RI components across studies (e.g., Christie et al., 2022; Garlovsky et al., 2024; Hernández-Hernández et al., 2021; Lowry et al., 2008), no consensus approach to classifying RI components has been widely adopted, either for individual systems or meta-analyses.

In 2021, we began collating RI data from case studies in many different clades to understand how RI evolves across the Tree of Life. Through this process, we identified four primary challenges in classifying individual estimates of RI. RI classifications must accommodate (1) the diversity of organismal life histories, (2) the differing resolutions at which RI estimates are made, (3) the nuances of postzygotic isolation, and (4) the multiple dimensions of RI. Building on the schema outlined by previous researchers and endeavoring to address these challenges, we propose a standardized framework to classify and categorize RI: the *Reproductive Isolation Ontology* (RIO). Here, we discuss how the RIO resolves the above challenges when classifying estimates of RI (Box 1), thus enabling us to create comparable datasets across diverse taxa to better understand the role of RI in speciation (Box 2 and Figure 1).

**Figure 1.**
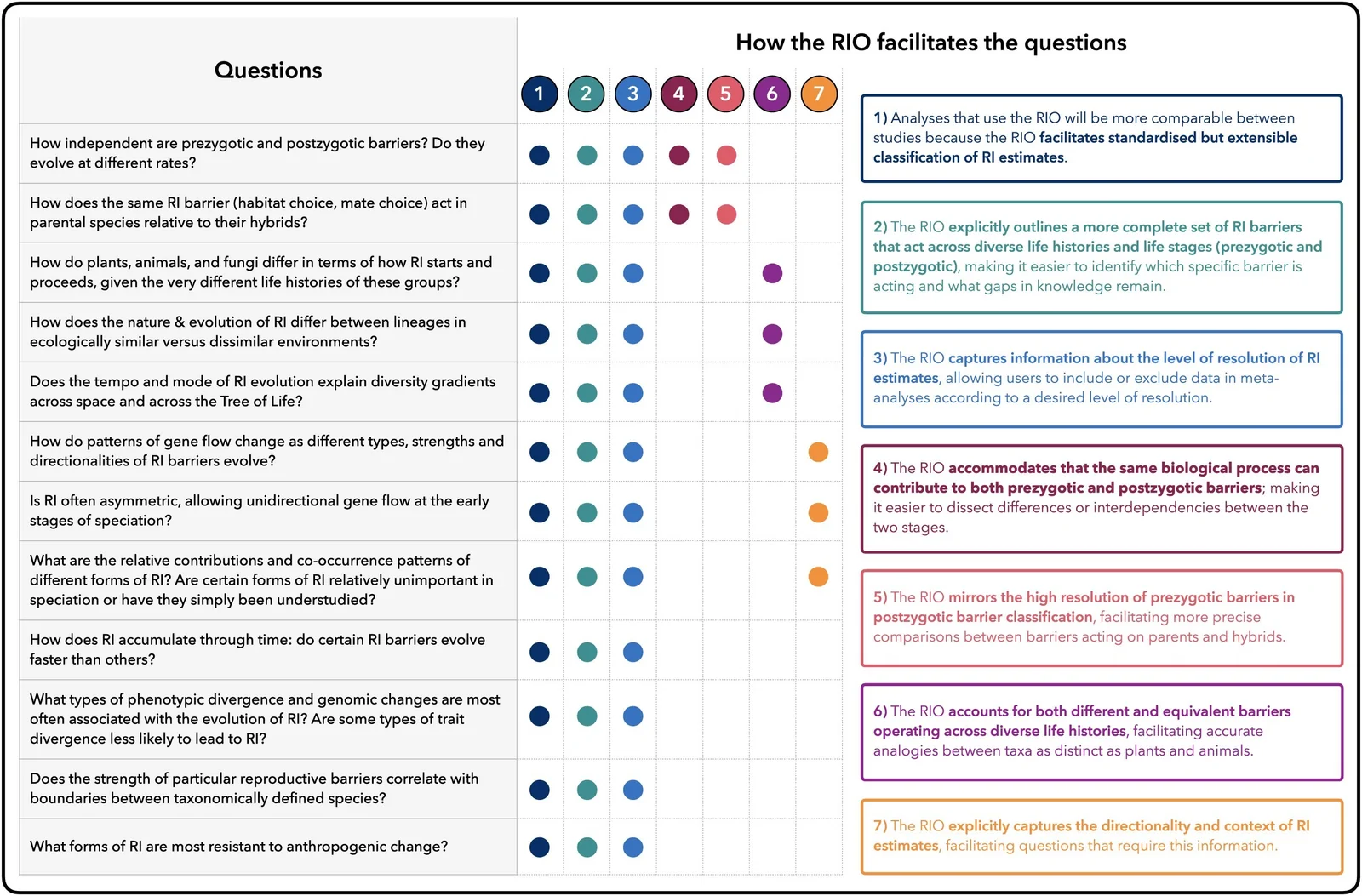
Questions about the role of reproductive isolation (RI) in speciation that the Reproductive Isolation Ontology (RIO) can help address.

Box 1: **Application of the Reproductive Isolation Ontology (RIO) to published studies**

***Example 1: Pollinator isolation in* Mimulus* monkeyflowers***

Ramsey et al. (2003) quantified pollinator isolation between *Mimulus lewisii* and *M. cardinalis* using observations of pollinator movements in natural sympatric populations. They recorded the number of pollinator “bouts” that involved a single pollinator visiting both species versus only one species and then used these data to estimate reproductive isolation (RI). With the RIO, we would classify this barrier as follows:

- *RI component:* This estimate would be classified as “biotic vector association” because it records the pattern of actual visits made by biotic vectors carrying pollen between the species.
- *RI context:* This estimate would be classified as “observation sympatric environment” because nonmanipulative observations were made in natural sympatric populations.
- *RI direction:* This estimate would be classified to the lowest resolution term “isolation,” because the direction of pollen transfer (i.e., *cardinalis → lewisii* or *lewisii → cardinalis*) was not recorded.

***Example 2: Reduced hybrid fitness in* Henosepilachna *ladybirds***

Matsubayashi and Katakura (2009) used controlled laboratory crosses to test for reduced fitness in F_1_ offspring between two species of phytophagous ladybird beetles, *Henosepilachna vigintioctomaculata* (“Hv”) and *H. pustulosa* (“Hp”). They combined five measures of performance—larval survival, adult survival, hibernation survival, mate choice success, and cross-fertility—into a single fitness estimate for each of the two reciprocal classes of F_1_ (*Hp*-mother/*Hv*-father and *Hp*-father/*Hv*-mother). They then compared these estimates to the performance of nonhybrid offspring. With the RIO, we would classify these barriers as follows:

- *RI component:* They can only be classified at low resolution to “postzygotic,” because multiple postzygotic barriers have been combined into a single postzygotic index of fitness. To have estimates classified at a higher resolution, they would need to separate the contributions of survival (“F1 hybrid inviability”), mate choice success (“F1 pairing association”), and cross-fertility (“F1 cross-fertility”).
- *RI context:* The estimates are classified as “experiment ex-situ” because performance assays were conducted in a nonnatural lab setting.
- *RI direction:* Because of the reciprocal design, the measurements associated with the different F1 classes generate estimates with different directionalities. Specifically, the barrier strength could be estimated by comparing the performance of either (i) *Hp*-mother/*Hv*-father F1s or (ii) *Hv*-mother/*Hp*-father F1s against the performance of either (a) *Hp* nonhybrid offspring or (b) *Hv* nonhybrid offspring. This generates four permutations for calculating RI, each representing a different directionality.

*See Table S1 for further examples*.

## Challenges of classifying RI estimates within existing schemes

Most modern classifications of RI are modifications of the structure originally proposed by Dobzhansky (1937b). This envisions RI as a sequence of successive barriers (“RI components”), each of which progressively reduces the likelihood of successful reproduction (Figure 2A). For example, consider two plant lineages with low levels of cross-pollination because they occur in largely non-overlapping geographic ranges. When cross-pollination does occur, hybrids rarely form because gametes are incompatible. The few hybrids that do form have low survival rates because their physiology is mismatched to their environment. The strengths of these three barriers could be estimated empirically and would be classified as “geographic isolation,” “fertilization isolation,” and “hybrid inviability,” respectively (Figure 2A). While this basic structure has facilitated decades of fruitful RI research, four key complications make it challenging to classify and compare RI estimates with this approach.

**Figure 2.**
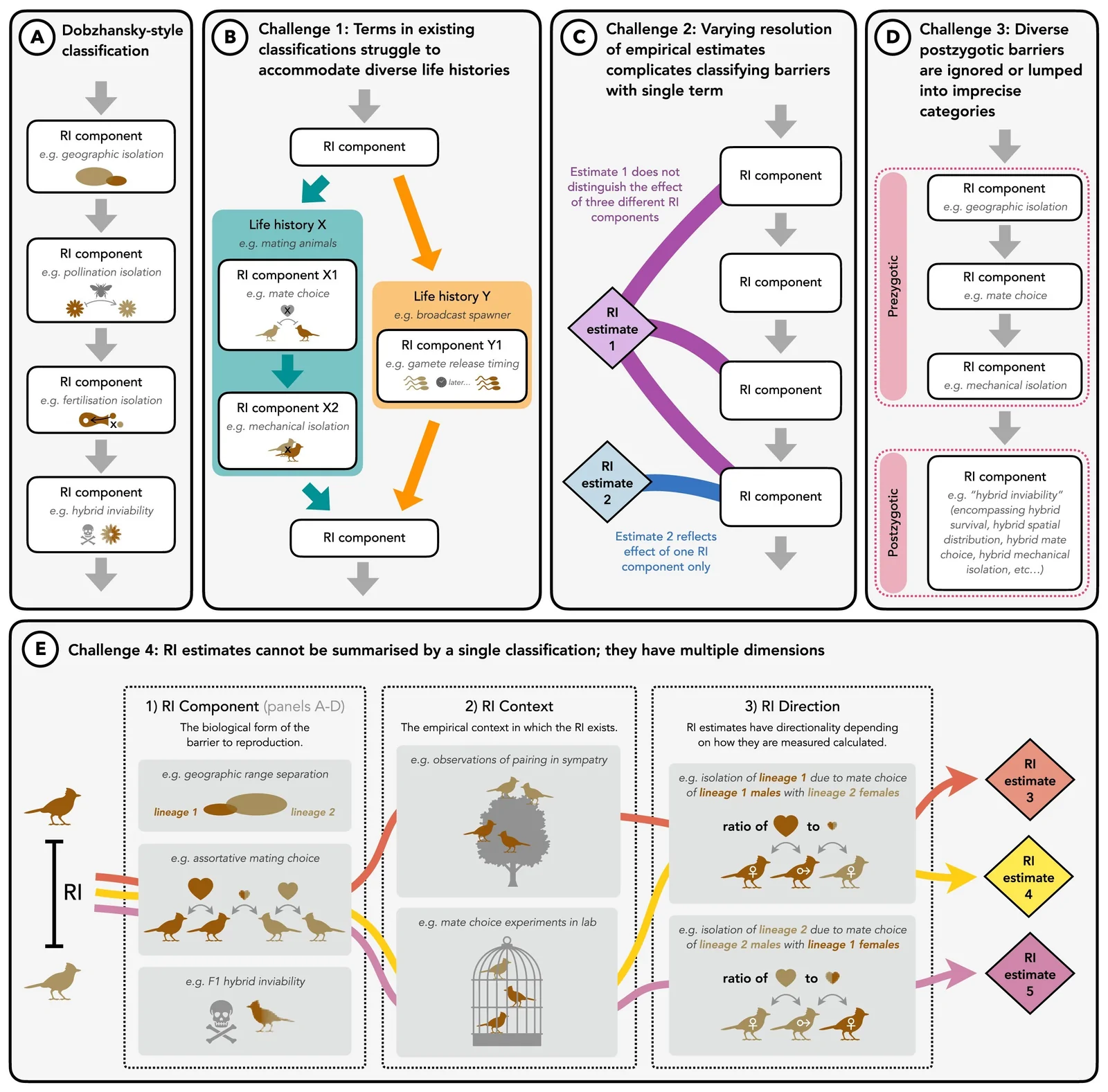
The current basic structure for classifying reproductive isolation (RI) and its complications. (A) Most existing schemes envisage RI as a sequence of successive barriers, each of which progressively acts to reduce the likelihood of reproduction. RI estimates would be classified by selecting the most appropriate barrier in this sequence (e.g., “geographic isolation”). This schema faces several complications: (B) existing barrier classifications cannot be easily applied across a diversity of life histories; (C) varying resolution of estimates makes it challenging to classify them with a single term; (D) diverse postzygotic barriers are typically overlooked or “lumped” into imprecise categories; and (E) RI has multiple dimensions beyond the barrier component and its strength.

### Challenge 1: Existing classifications of RI exclude some organisms

Most existing classifications are designed around a typical animal or plant life cycle, in which sexually reproducing individuals are born, generate and exchange gametes, and produce offspring by the fertilization of these gametes. Many organisms do not follow this pattern or differ in the details of those stages (Figure 2B), meaning that existing classifications may force barriers to be described with terms that are inaccurate. For instance, plants and broadcast-spawning animals (e.g., corals) do not “mate” but instead disperse their gametes/pollen, so terms like “pre-mating” and “post-mating” are somewhat inappropriate. Similarly, some classifications include highly taxon-specific terms like “sperm competition” or “pollen-pistil interactions” that lack clearly defined equivalents in other taxonomic groups. At the same time, creating unique classifications for every taxon with a slightly different reproductive cycle would limit our ability to compare across genuinely analogous barriers in these taxa (e.g., temporal barriers, or wind pollination in plants and broadcast spawning in animals). We therefore need a classification that simultaneously (i) accommodates a wide range of life histories with accurate terminology, (ii) meaningfully groups equivalent barriers together across taxa, and (iii) avoids lumping nonequivalent barriers together.

### Challenge 2: Varying resolution complicates classifying barriers within a single term

In existing schemes, each RI estimate is classified as a single component within a clear chronological sequence (Figure 2A). However, estimates of RI strength cannot always be easily ascribed to a single component (Figure 2C). As an example, take the case of two plant lineages that rarely produce hybrid seed. The low yield of hybrid seed could be caused by pollen rejection (a prezygotic barrier that acts after the transfer of gametes) and/or low viability of hybrid embryos (a postzygotic barrier). A study that only measures the relative yield of hybrid versus nonhybrid seed cannot distinguish between these two barrier components of RI and would need an RI classification that reflects this uncertainty. In contrast, a more complex study that estimates the separate roles of pollen rejection and embryo viability in reducing hybrid seed yield could apply a more precise RI classification (Garlovsky et al., 2024).

As another example, consider two bird species adapted to different altitudes. Suppose that a high-altitude individual ends up at low altitude and hybridizes with an individual from a low-altitude species. The resulting hybrid offspring have low survival, which could simply be recorded as “hybrid inviability.” But imagine further that this low survivorship is known to be partially due to reduced parental provisioning by the high-altitude parent struggling with the heat of the low-altitude habitat. The ecological maladaptation of parents exists before the formation of hybrid offspring and is a form of “immigrant inviability”, but the reduced provisioning rate only manifests after fertilization through the poor performance of a hybrid offspring. An empirical estimate of this barrier could not be neatly ascribed to a single RI term due to the barrier’s complexity and the fact that it results from both prezygotic and postzygotic factors. Further experimental work would be required to separate the different contributions of “immigrant inviability” and “hybrid inviability,” thus increasing the resolution of the RI estimate.

These two examples show how experimental limitations and the complexity of RI barriers can result in variation in the resolution at which they are recorded. Moreover, even if researchers measure RI at higher resolution, they may also choose to merge estimates of different barriers in order to estimate “total isolation,” “prezygotic isolation,” or “postzygotic isolation.” Comparing RI components across diverse studies thus requires a classification system that accounts for different levels of resolution to compare like with like. Developing a method of RI classification that explicitly accommodates different levels of resolution would enable a more functional and inclusive description of empirical estimates of RI.

Box 2: **Case study: How would the Reproductive Isolation Ontology (RIO) change a comparative study?**

A common language for both quantifying and classifying estimates of reproductive isolation (RI) allows us to turn heterogeneous data points into comparable ones that can facilitate broad-scale analysis. As an example, let us revisit the classic question about the relative rates at which prezygotic and postzygotic RI evolve (Coyne & Orr, 1989). Matute and Cooper (2021) identified only 10 studies across 10 clades that addressed this question; we know of one other relevant study (a meta-analysis in plants) published since their 2021 review (Christie et al., 2022). Thus, although this question is important and seemingly simple, it is relatively untested. Yet, a literature search suggests that nearly 1,000 papers have been published that measure prezygotic and postzygotic isolation, suggesting our field should be able to test this hypothesis more completely if we can harness the information in these published studies. How might we do so?

***Without the RIO***

Following a literature search, we would extract RI barrier type and RI strength from many studies. Unless we implemented a finer resolution scheme for RI like that used in Christie et al. (2022), we would classify different barriers coarsely as either prezygotic or postzygotic based on the authors’ descriptions. We would additionally survey the literature to associate RI components with metadata like divergence time between lineages and their geographic origins. Ultimately, we would regress prezygotic and postzygotic RI strength against divergence time to address our question. As Matute and Cooper (2021) showed, patterns are likely to vary across clades. But the coarseness of our dataset would make it challenging to understand the underlying causes. In the future, researchers revisiting this analysis would need to reconstruct our classification scheme to expand or refine the dataset.

***With the RIO***

Following a literature search, we would extract RI estimates from studies. Some researchers would have associated RIO terms with their estimates, expediting our review; we would be able to classify the remaining studies in a standardized and reproducible way using the RIO. After collecting metadata, the nested hierarchy of the RIO would allow us to collapse all RIO terms to the broadest resolution: “prezygotic” versus “postzygotic.” We would then analyze these prezygotic and postzygotic measurements against divergence time to answer the question. If we found unexpected results, we could use the additional data embedded in the RIO to understand why—for example, is the biological basis of prezygotic barriers also reflected in the biological basis of postzygotic barriers? Do asymmetries in prezygotic versus postzygotic isolation affect the rate at which they evolve? Later, because the RIO has created a standardized language that makes sharing of RI data across studies easier, researchers will be able to extend these analyses by augmenting this dataset with new studies and/or analyzing this dataset in new directions. Thus, the RIO ensures that this painstaking work of extracting data from hundreds of published papers is more interoperable and more reusable for future researchers.

### Challenge 3: Diverse postzygotic barriers are often ignored or lumped under imprecise terms

Prezygotic barriers are typically finely subdivided. For example, researchers regularly distinguish between habitat, temporal, pollinator, behavioral, and mechanical components of prezygotic RI (Coyne & Orr, 2004; Dobzhansky, 1937b; Mayr, 1963). By contrast, postzygotic barriers are typically lumped into a few broad categories—i.e., “hybrid sterility” or “hybrid inviability”—even though postzygotic barriers mirror prezygotic barriers in their number and diversity (Figure 2D). For example, a hybrid might be effectively sterile for multiple reasons, including phenological divergence that reduces overlap in breeding season (a form of temporal isolation) or morphological divergence in reproductive organs that impairs successful copulation (a form of mechanical isolation). Further, under some existing schema, these barriers would be classified as prezygotic—even though they act on the hybrid—and would be lumped together with barriers acting on parental species.

This lack of precision has hampered our ability to distinguish among fundamentally different forms of postzygotic isolation. As a result, certain components of RI are under-represented or remain largely unexplored, preventing us from addressing fundamental questions about the evolution and nature of postzygotic isolation (Matute & Cooper, 2021). To overcome these limitations, a revised classification of RI would ideally expand the terminology used for postzygotic barriers to mirror the precision typically used for prezygotic barriers, with comparable levels of resolution. This classification will allow us to understand how parental barriers can be broken or reshaped in hybrids, thus influencing hybrids’ ability to act as a bridge to gene flow between parental species.

### Challenge 4: RI estimates have multiple dimensions

Empirical estimates of RI often focus on the specific reproductive barrier they are measuring and its strength. However, RI estimates have other important dimensions that provide context about these barriers and are essential for their accurate interpretation (Figure 2E). For example, RI can be asymmetric, such that one lineage will more readily or successfully reproduce with, and receive alleles from, another lineage than vice versa. Successful reproduction may also be more likely when the female belongs to one lineage rather than the other. Asymmetry in RI is expected under several scenarios, including the evolution of unidirectional genetic incompatibilities (Turelli & Moyle, 2007) or as a result of reinforcement selection (Yukilevich, 2012). Thus, capturing the directionality of RI is important.

Additionally, RI is measured in different environments, such as in the laboratory, under common garden greenhouse conditions, or in the wild. The strength of the same barrier might differ across environments (Rundle, 2002), so RI strength should be classified relative to the environment in which it is measured. Thus, a revised classification of RI should capture the multiple dimensions of RI.

## Our proposal: an ontological classification for RI estimates

Our goal is to provide a precise but flexible framework for classifying estimates of RI that addresses the challenges outlined above. Many biological data, including estimates of RI, can be described both in terms of the biological phenomena they quantify and the relationship of those phenomena to one another. An ontology provides a formal way to structure such data, using a controlled vocabulary to classify individual data points and link them through explicit relationships between the terms in the vocabulary. For example, the widely used Gene Ontology (GO) classifies a gene’s function using three separate domains: “biological process,” “molecular function,” and “cellular component.” Each domain is a hierarchy of controlled terms; higher-resolution terms are nested within broader ones. For example, “biological process” contains the broad term “gene expression,” within which the more specific processes “RNA processing” and “mRNA splicing” are nested. The ontology offers two primary benefits. First, the standardized language allows users to identify comparable data points across studies. Second, the hierarchical nature of the ontology allows data points to be classified to the appropriate level of resolution, in accordance with our existing knowledge. The nested hierarchy allows researchers to collapse or filter results to a desired resolution, further facilitating comparative analysis.

Taking inspiration from the GO, we propose the Reproductive Isolation Ontology (“RIO”; Figure 3). The RIO comprises controlled vocabularies in three domains—RI component, RI direction, and RI context—each of which classifies a different attribute of single RI estimates, as discussed below (see the Supplementary Material for a full description of all current RIO terms). While we believe that these three domains and their vocabularies capture the most salient features and forms of RI estimates, the RIO is extensible and can be expanded to include new terms across these three domains and/or new domains. In practice, any estimate of RI strength can be classified by selecting one term from each of the domains (see Box 1 and Table S1 for worked examples).

**Figure 3.**
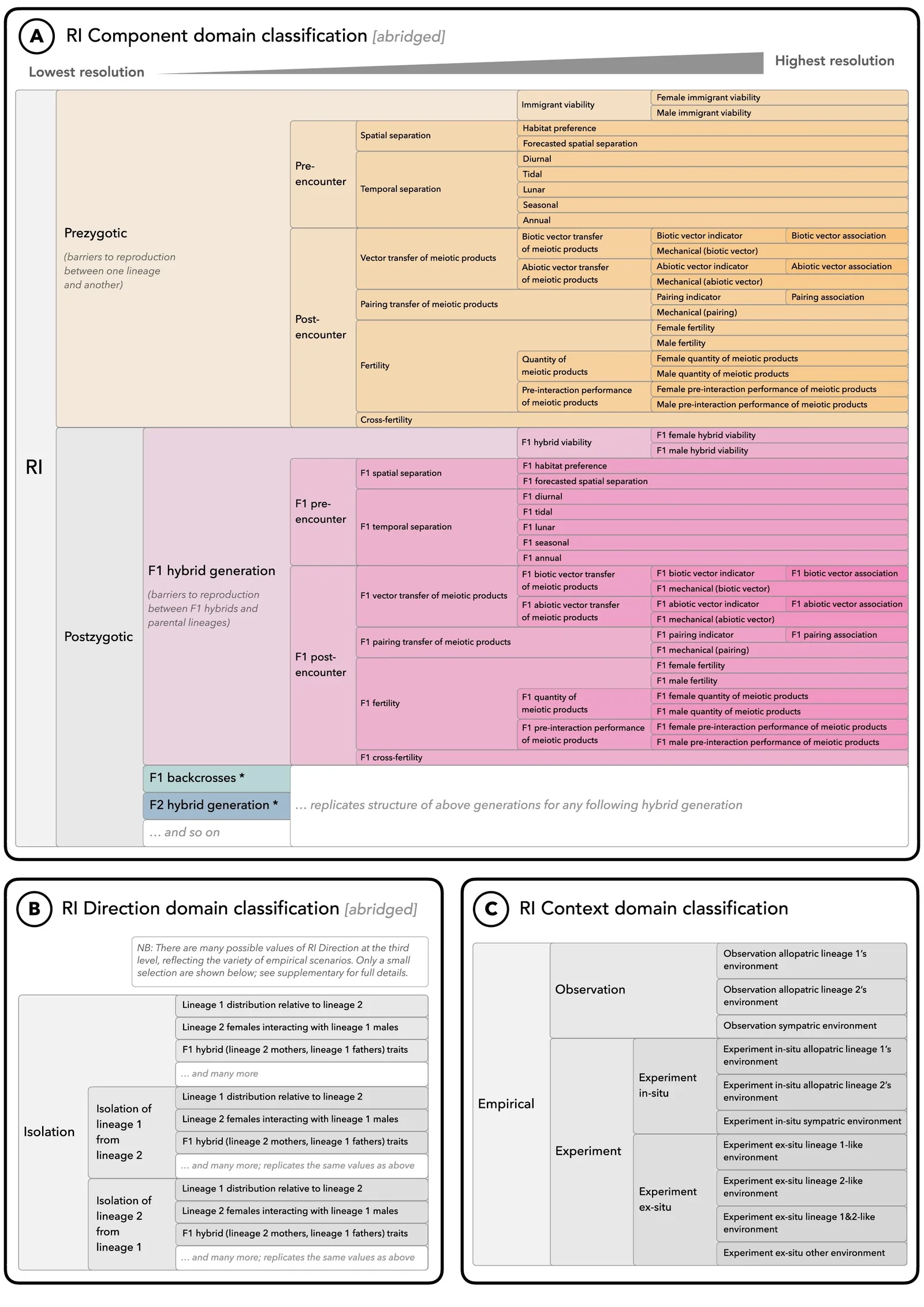
The Reproductive Isolation Ontology (RIO) consists of three classifications or “domains”: (A) “component,” or the type of barrier to reproduction; (B) “direction,” or the directionality of the barrier measurement; and (C) “context,” or the nature of the experimental and observational study used to measure the barrier. The hierarchical structure of the RIO allows researchers to identify the appropriate resolution for their study.Supplementary Material outlines the full terms included in the RIO. * For simplicity, the first version of the RIO only accommodates postzygotic RI measurements that involve F1s, but future versions can accommodate a greater range of hybrid generations.

### RI component

The RI component domain (Figure 3A; Figure S1 and Table S3) classifies an RI estimate according to the biological basis of the isolating barrier. It has been designed to offer standardized language for RI components that accommodates a diversity of life histories, to allow differing levels of study resolution, and to capture all the nuances of postzygotic isolation.

Many existing RI classifications already implicitly group different RI barriers into hierarchies—for example, temporal, behavioral, and habitat isolation are all nested within prezygotic isolation, and hybrid sterility and hybrid inviability are specific types of postzygotic isolation (Coyne & Orr, 2004). Here, we build on this foundation to create a more explicit and detailed nested hierarchy. For example, most existing classifications categorize temporal isolation as a form of prezygotic isolation, as we do here. The RIO further dissects prezygotic isolation into types that act solely before lineages interact (“pre-encounter”)—among which temporal isolation is included—and those that act solely after lineages interact (“post-encounter”). “Immigrant viability,” whose effect is observed both before and after a reproductive encounter, is placed outside this division. Additionally, we categorize temporal isolation more finely—i.e., species can be isolated because they keep different “seasonal” or “diurnal” schedules. This more detailed hierarchy better captures the diversity of RI barriers seen in nature.

Critically, because this hierarchy is nested, it can accommodate differing levels of resolution across different RI estimates (cf. Challenge 2). Imagine a system in which two insect species rarely mate because they rarely co-occur in the same habitat. Researchers might use habitat choice tests and find that the species choose to associate with different host plants, reducing rates of encounter (“habitat preference” in the RIO); or they might use spatial distribution modeling to forecast broad-scale geographic ranges of the two species using climate data to determine how much they overlap (“forecasted spatial separation”). If they had measured a less precise pattern—e.g., simply drawn polygons around their observed occurrences and calculated the proportional range overlaps—this would be classified with a lower resolution term in the RIO: “spatial separation.” This nested hierarchical structure—combined with the unique alphanumeric codes for each term (Table S3)—will also facilitate meta-analyses; researchers will be able either to select only those studies conducted at the relevant level of resolution or to collapse studies down to more coarse categories, as required.

Additionally, we have worded RI component terms carefully to accommodate a diversity of reproductive life histories (cf. Challenge 1; see Glossary). First, we have identified barriers and life stages in diverse taxa that are comparable and have generated taxon-agnostic terms that allow them to be classified together. For example, the life stage between meiosis and fertilization is typically referred to as “gametes” in animals, “spores” in many sexual fungi; and for sporophyte-dominant plants, this life stage encompasses spores, gametophytes (e.g., pollen and ovule), and gametes. We therefore use the taxon-agnostic term “meiotic products” to encompass this diversity of terms in the RIO. This more generalized terminology will facilitate comparisons of RI components that act on comparable life stages but in different taxa. At the same time, we recognize that some barriers and life stages are not comparable in different taxa because they experience different selective pressures and therefore require RI components to be classified separately. For example, some plants rely on wind or insects for cross-pollination, some broadcast-spawning animals similarly rely on ocean currents for cross-fertilization, and many animals require mating for cross-fertilization. Because some of these systems are decidedly distinct, we distinguish them as “abiotic vector transfer of meiotic products” (e.g., wind-pollinated plants and broadcast-spawning animals), “biotic vector transfer of meiotic products” (e.g., insect-pollinated plants), and “pairing transfer of meiotic products” (e.g., animals that mate). More broadly, our choice of language ensures that diverse estimates of RI are classified accurately and can be compiled in syntheses.

As currently framed, the RIO only accommodates sexual life histories, thus excluding all prokaryotes and some eukaryotes. It further assumes that nuclei fuse following fertilization to form a diploid (or higher-ploidy) organism that is the dominant life stage, relative to the haploid tissues produced by meiosis. The RIO therefore currently excludes some fungi in which meiotic products fuse but their nuclei do not, as well as bryophytes and many algae in which the haploid stage is dominant. However, unifying the classification of RI estimates for many sexual eukaryotes (especially those for which RI data typically exist) is a concrete first step, and we hope to expand its applicability in future.

Finally, RIO expands the postzygotic terms in the RI component classification to mirror the prezygotic terms, enabling postzygotic estimates of RI to be classified to the same precision as prezygotic estimates (cf. Challenge 3). For example, estimates of mate choice between two animal lineages would be categorized as the prezygotic term “pairing association.” If we then measure mate choice of F1 hybrids relative to their parents or to each other, we can categorize those estimates as the postzygotic term “F1 pairing association.” We hope this finer dissection of terms will stimulate research on more overlooked components of postzygotic isolation. The RIO is currently designed to accommodate only F1 hybrids; interactions involving later-stage hybrid classes cannot be accommodated at present (though we aim to add this in future versions). However, the flexibility of the RIO means (1) if a hybrid lineage is stable, then it can be represented as a parental lineage in the RIO and its interactions can be catalogued and (2) future researchers can expand the RIO to be more inclusive of other hybrid classes.

### RI direction

The RI direction domain (Figure 3B; Figure S2 and  Table S5) refines an RI estimate according to its directionality. Asymmetries in RI between pairs of lineages are expected to arise from multiple sources (Orr, 1997; Turelli & Moyle, 2007; Yukilevich, 2012). By capturing the directionality of RI, we will be better able to understand how asymmetry in RI evolves and impacts lineage divergence (cf. Challenge 4). The lowest level of resolution in the RI direction ontology is an estimate without any directionality (i.e., “the average isolation of species A and species B from each other”). At higher resolution, RI direction distinguishes estimates based on the identity and sex of the interacting lineages. For example, the temporal “isolation of species A from species B” versus the “isolation of species B from species A” would be different in the case where species A has a short reproductive period that is completely contained within the longer reproductive period of species B. Many of the values of RI direction listed in Table S5 currently only accommodate organisms with male and female individuals/tissues, and we hope to expand to other scenarios (including mating types) in future.

### RI context

The RI context domain (Figure 3C; Table S4) both categorizes how RI was measured—i.e., whether by observation, experiment in a controlled setting, or experiment in a natural habitat—and how the environment relates to the natural environments of the lineages under study. The strength and direction of RI may vary depending on the environment in which it is measured (Hatfield & Schluter, 1999; Rundle, 2002), and understanding when RI is dependent on the environment might help us predict the stability of species barriers in changing environments. RI direction and RI context together allow a more holistic classification of RI, providing greater context to our understanding of RI composition, strength, and evolution (cf. Challenge 4). Like the terms for RI components, these domains are organized in a nested hierarchy using terms that should work for a diversity of taxa.

## Applications, adoption, and outlook of the RIO framework

The RIO (Figure 3) provides a standardized language for categorizing RI, and it represents an additional effort to share RI data more broadly. Combined with advances in standardizing the calculation of RI strength (Sobel & Chen, 2014), the RIO will help us bring together RI data from diverse case studies to better understand speciation (Figure 1 and Box 2).

Like any general framework, the RIO obscures many interesting biological details, including the interdependence and interactions among different categories of RI. This simplification is necessary to achieve broad comparability of terms. But, like most ontologies, the RIO is flexible and extensible and thus can evolve to meet the needs of the speciation community. For example, although we have focused on organismal measures of RI, additional domains could be developed to accommodate genetic measures of RI, such as those derived from demographic models (De Jode et al., 2023) or genome scans for local barrier effects (Laetsch et al., 2023). Similarly, Sobel and Chen (2014)’s equations for quantifying the strength of RI from empirical data are now widely used, but other equations have also been proposed, new approaches will almost certainly be developed in future, and all of these can easily be accommodated by the RIO. Further, we can achieve even greater integration across speciation studies by standardizing the reporting of metadata associated with RI estimates, such as the nature and degree of ecological and genetic divergence.

The long-term utility of the RIO will depend both on its application retrospectively to the wealth of existing RI datasets, and on its adoption in future studies. Other biological ontology initiatives (such as the GO) have succeeded when the community has recommended modifications where required and adopted using the ontology as a normal part of research culture (Bard & Rhee, 2004). We therefore encourage speciation researchers to both engage with the RIO and report their RI data with associated RIO terms. We invite the research community to learn more about the RIO, provide feedback, and suggest modifications. The IOS network website *https://speciation-network.pages.ist.ac.at/resources/* will maintain an up-to-date guide and dictionary of the RIO for future researchers to adopt (at time of publishing, this is the Supplementary Material). We also plan for it to host a user-friendly guided decision tree that enables researchers to classify their data within the RIO without requiring a comprehensive knowledge of all its intricacies.

Ultimately, using the RIO will help researchers to adhere to the FAIR principles of data stewardship, which call for data to be findable, accessible, interoperable, and reusable (Wilkinson et al., 2016). By reporting RI data with RIO terms, researchers will ensure that their findings can be properly interpreted, included in large meta-analyses, and ultimately contribute toward answering enduring questions about the origins of biodiversity.

**Table 1.** Glossary of key terms (additional detail can be found in the RIO glossary, Table S2)

Reproductive isolation (RI)	The extent to which reproductive barriers between a pair of lineages reduce the likelihood of successful reproduction between them relative to within them.
Lineage	The evolutionary unit of comparison (including species, subspecies, varieties, morphs, ecotypes, and cytotypes). RI is measured between pairs of lineages.44
Component of RI	A feature of an organism’s biology that generates a reproductive barrier, and thus RI, between lineages. Components of RI come in diverse forms, including prezygotic and postzygotic.
Ontology	A hierarchical classification of something, usually associated with a controlled vocabulary, in which values are nested within parent values, which are nested within their own parent values, and so on. Ontologies often contain multiple “domains,” with each domain being its own ontological classification of a different feature of the thing being classified.
Meiotic products	The parts of an organism’s life cycle after meiosis and up until fusion of gametes to form a zygote. For sexual animals, the “meiotic products” are gametes (typically sperm and eggs). For sexual sporophyte-dominant plants, the “meiotic products” are spores, gametophytes (including pollen and ovule), and gametes.
Vector transfer of meiotic products	A step in some life cycles when meiotic products are transferred from one individual to another via a third organism (e.g., an insect pollinator) or via an abiotic agent (e.g., via wind or ocean current). For example, in plants, this consists of pollen being carried by the wind/pollinator to female reproductive tissues. In broadcast-spawning animals, this consists of gametes being released into the environment where they meet other individuals’ gametes.
Pairing transfer of meiotic products	A step in some life cycles when individuals select other individuals with whom they “pair up” and directly exchange meiotic products. For example, in animals with internal fertilization, this consists of mate preference and mating. In animals with external fertilization between breeding pairs, this consists of mate preference and gamete mixing.
Female/male	For the purposes of the RIO, we use “female” to describe egg-producing individuals and tissues and “male” to describe sperm-producing individuals and tissues. For example, for dioecious plants, female individuals produce ovules and eggs, and male individuals produce pollen and sperm. For monoecious plants, an individual contains female and male tissues.

## Supplementary Material

qrag023_Supplemental_File

## Data Availability

An up-to-date guide and dictionary of the RIO will be maintained at: https://speciation-network.pages.ist.ac.at/resources/.
